# Providers’ Perceptions of Parental Human Papillomavirus Vaccine Hesitancy: Cross-Sectional Study

**DOI:** 10.2196/13832

**Published:** 2019-07-02

**Authors:** Jennifer Cunningham-Erves, Tatsuki Koyama, Yi Huang, Jessica Jones, Consuelo H Wilkins, Lora Harnack, Caree McAfee, Pamela C Hull

**Affiliations:** 1 Department of Internal Medicine Meharry Medical College Nashville, TN United States; 2 Department of Biostatistics, Vanderbilt University Medical Center Nashville, TN United States; 3 Division of Epidemiology, Department of Medicine, Vanderbilt University Medical Center Nashville, TN United States; 4 Meharry-Vanderbilt Alliance Vanderbilt University Medical Center Nashville, TN United States; 5 Cumberland Pediatric Foundation Nashville, TN United States

**Keywords:** neoplasms, papillomavirus infections, papillomavirus vaccines, primary prevention, health care provider, vaccine hesitancy, provider barriers to HPV vaccination

## Abstract

**Background:**

Human papillomavirus (HPV) vaccine hesitancy among parents contributes to low vaccination coverage in adolescents. To improve health care provider communication and vaccine recommendation practices with hesitant parents, it is important to understand how providers perceive parental HPV vaccine hesitancy.

**Objective:**

This study aimed to characterize perceived reasons for parental HPV vaccine hesitancy and identify factors associated with perceived parental hesitancy among providers at community-based pediatric clinics.

**Methods:**

In 2018, providers in 23 community-based pediatric clinics in Tennessee were invited to complete a Web-based baseline survey as part of a larger quality improvement study focused on HPV vaccine uptake. These survey data were used for a cross-sectional, secondary data analysis. Scale scores ranging from 0 to 100 were calculated for provider self-efficacy (confidence in ability to recommend HPV vaccine), provider outcome expectations (expectations that recommendation will influence parents’ decisions), and perceived parental HPV vaccine hesitancy. Provider confidence in HPV vaccine safety and effectiveness were categorized as high versus low. Clinic-level exposures examined were clinic size and rural-urban location. Descriptive analyses were used to characterize perceived parental barriers by provider type. Mixed-effects linear regression models were fit taking one exposure variable at a time, whereas controlling for provider type, age, gender, and race to identify provider- and clinic-level factors associated with perceived parental barriers to HPV vaccination.

**Results:**

Of the 187 providers located in the 23 clinics, 137 completed the survey. The majority of physician providers were white and female, with a higher percentage of females among nurse practitioners (NPs) and physician assistants (PAs). The most common parental barriers to HPV vaccination perceived by providers were concerns about HPV vaccine safety (88%), child being too young (78%), low risk of HPV infection for child through sexual activity (70%), and mistrust in vaccines (59%). In adjusted mixed models, perceived parental HPV vaccine hesitancy was significantly associated with several provider-level factors: *self-efficacy* (*P*=.001), *outcome expectations* (*P*<.001), and *confidence in HPV vaccine safety* (*P*=.009). No significant associations were observed between perceived parental HPV vaccine hesitancy and clinic-level factors *clinic size* nor *location*.

**Conclusions:**

Researchers developing provider-focused interventions to reduce parental HPV vaccine hesitancy should consider addressing providers’ self-efficacy, outcome expectations, and confidence in HPV vaccine safety to help providers communicate more effectively with HPV vaccine hesitant parents.

## Introduction

### Background

Human papillomavirus (HPV) vaccination coverage remains alarmingly low. In 2017, only 49% of adolescents aged 13 to 17 years in the United States completed the recommended doses of the HPV vaccine [1]. These rates fall short of the national goal of 80% coverage by 2020 for HPV vaccination of adolescents aged 13 to 17 years [2]. This warrants great concern as the effects of HPV infection remain high and many are at risk of HPV-associated cancers [3]. Parental vaccine hesitancy toward the HPV vaccine is a major contributor to low uptake of the vaccine and a growing public health problem [4-6]. According to a national survey, at least one-third of families are vaccine hesitant, meaning they delay or decline the HPV vaccine when initially recommended [7]. Therefore, understanding sources of parental hesitancy is important to develop strategies to address their concerns.

### Reasons for Parental Human Papillomavirus Vaccine Hesitancy

A growing literature has begun to explore HPV vaccine hesitancy among parents in recent years. Common reasons for HPV vaccine hesitancy reported by parents include misinformation, lack of or varying recommendation, lack of knowledge, and concerns about vaccine safety and side effects [7-10]. Improving the quality of provider-patient communication is a key strategy in addressing the needs of hesitant parents [11,12], given that routine provider recommendation is the most preferred approach among parents to influence HPV vaccine uptake [13,14]. A strong, high-quality recommendation should promote the importance of vaccine, demonstrate urgency, and emphasize cancer prevention [15]. According to a recent qualitative study with 43 HPV vaccine hesitant parents, providers with a persistent response in provider-patient interactions had higher rates of same-day vaccination compared with providers who did not [16]. A persistent response refers to a provider continuing discussion on the vaccine (ie, talking about its importance, providing a strong recommendation, and querying parental concerns on the vaccine) after a parent declines or expresses a desire to delay the vaccine [16].

### Parental Human Papillomavirus Vaccine Hesitancy and Provider and Clinic Level Factors

A previous study found a mismatch between provider and parental ratings of how much importance parents placed on HPV vaccine, with providers substantially overestimating the parental HPV vaccine hesitancy [17]. How providers perceive parental HPV vaccine hesitancy may determine their willingness to recommend the HPV vaccine, how they present the vaccine recommendation to parents, and how they respond when parents refuse the vaccine [18]. A few studies have explored what providers perceive as parents’ reasons for HPV vaccine hesitancy and how these perceptions correlate with HPV vaccination outcomes [19-21]. Thus, it is important to examine provider-level variation in how they perceive parental HPV vaccine hesitancy, given that many providers may not perceive parents’ level of hesitancy accurately and that their perceptions of parental hesitancy are associated with provider-level variation in HPV vaccination rates. Yet, research to date has not identified the factors that influence provider perceptions of parental HPV vaccine hesitancy. These factors could be targets for interventions to improve provider communication and recommendation practices.

A previous study found that routine provider recommendations for the HPV vaccine were more likely to occur with providers who had a high confidence in their ability to recommend the vaccine and address parental concerns (ie, high context-specific self-efficacy). Providers with high expectations of their recommendations resulting in parents accepting the vaccine for their children (ie, high outcome expectations) were also more likely to recommend the HPV vaccine routinely [20]. Another study found that providers with lower confidence in HPV vaccine efficacy and safety had lower HPV vaccine uptake among their patients [19]. These provider characteristics could also influence their level of perceived parental HPV vaccine hesitancy. These characteristics should also be considered by provider type as: (1) a recent study suggests approximately one-half of initial recommendations are given by providers who are not physicians (Malo et al, in press; [15]); and (2) parent-provider interactions may influence perceptions of physician versus nonphysician providers differently and how they recommend the vaccine.

Clinic-level factors could also potentially affect perceived parental HPV vaccine hesitancy. For example, rural areas have lower HPV vaccination coverage compared with urban areas [1]. Lower coverage in rural areas could be due to a combination of fewer or weaker provider recommendations, greater parental hesitancy, or both. If providers in rural areas perceive greater parental HPV vaccine hesitancy compared with those practicing in urban areas, they may be less likely to recommend the vaccine strongly to avoid disagreements with parents. One study has shown that smaller clinics tend to provide a more *personal* experience and have fewer changes in doctors, whereas another indicated preventive service (eg, childhood immunizations) was more apt to be delivered by larger clinics [22]. Therefore, the size of clinic could influence the length and type of patient-provider interactions, which indirectly affects providers’ perceived parental HPV vaccine hesitancy. However, no studies to our knowledge have identified specific factors that influence the types and level of parental HPV vaccine hesitancy that providers perceive and whether those factors vary by provider type.

The aim of this study was to characterize the reasons for and level of parental HPV vaccine hesitancy as perceived by pediatric providers at community-based, private pediatric clinics in Middle Tennessee. This study also aimed to identify provider-level and clinic-level factors influencing perceived parental hesitancy according to providers. The research question was: “What are the provider and clinic characteristics associated with perceived parental hesitancy among pediatric providers within community-based pediatric clinics in Middle Tennessee, surveyed from January to March 2018?” We hypothesized that perceived HPV vaccine hesitancy would be higher among providers who have lower self-efficacy, lower outcome expectations, lower confidence in HPV vaccine safety, and lower confidence HPV vaccine effectiveness. We also hypothesized that perceived HPV vaccine hesitancy would be higher among clinics that were larger and are located in small towns that serve rural areas. Study findings can be used to develop interventions that assist providers in effectively engaging HPV vaccine hesitant parents to improve acceptance and vaccination outcomes.

## Methods

### Study Design and Data Source

This cross-sectional study used secondary data from 137 health care providers who provide care in 23 community-based pediatric clinics in Middle Tennessee. These providers are a part of an ongoing quality improvement parent study designed to compare the clinical effectiveness and cost effectiveness of 2 approaches to delivering quality improvement coaching focused on HPV vaccination, namely, Web-based coaching versus in-person coaching. As part of that parent study, providers completed a baseline survey that was collected from January to March 2018. This survey asked the providers questions related to HPV vaccine uptake in their clinics, their perceptions and attitudes related to HPV vaccine (eg, perceived barriers, self-efficacy, and outcome expectations), and demographic characteristics of providers. Clinic location (rural/urban) was determined based on the clinic address and clinic size was reported by the clinic. For this study, we analyzed data from the baseline provider survey data. This study was approved by Meharry Medical College and Vanderbilt University Institutional Review Boards.

### Study Population

The population was composed of providers from 23 private, community-based pediatric practices located across the Middle Tennessee Region that were members of Cumberland Pediatric Foundation (CPF). As a nonprofit organization, CPF applies scientific, charitable, and educational approaches to improve health care services for children. The foundation currently serves approximately 700 physicians, 70 practices, and 40 counties [23]. Practices were recruited for the parent study at events held by CPF or face-to-face by the research team. After practices made a practice-level decision to be in the trial, the providers were asked to take part in the survey. Providers included pediatricians, NPs, and PAs. The study inclusion criteria included all providers at each clinic, male or female, as they all provide HPV vaccines. None of the providers in the study clinics were excluded.

### Independent Variables

#### Self-Efficacy

Adapted from McRee et al (2015) [20], the self-efficacy measure assessed providers’ perceived confidence in their ability to recommend HPV vaccine and address parents’ concerns. It was composed of 6 items using a 5-point Likert scale based on the level of agreement (ie, strongly disagree to strongly agree). Example items included “I was confident I could explain the benefits of HPV vaccination to parents” and “I was confident I could overcome parental concerns about HPV vaccine safety.” Before the analysis, the scores of the items responses were recoded using a range of 0 to 100 with 0=strongly disagree, 25=disagree, 50=neutral, 75=agree, and 100=strongly agree, so that there would be a comparable numerical range across all the scales for ease of interpretation (ie, standardization of the variables), given that the outcome variable was measured on a 4-point scale. Higher scores indicated greater levels of self-efficacy. McRee et al [20] developed the items using cognitive interviews with health care providers but did not report on psychometric properties of the scale. As there were no other validated measures for self-efficacy, we used this measure. In our sample, the self-efficacy scale demonstrated good internal consistency with Cronbach alpha=.79.

#### Outcome Expectations

The outcome expectations measure, adapted from McRee et al (2015) [20], assessed providers’ expectations for whether their parental discussions lead to vaccination. It was composed of 4-items using a 5-point Likert scale based on the level of agreement with specific statements (ie, strongly disagree to strongly agree). “I was usually able to convince hesitant parents to get the HPV vaccine” and “When parents wished to delay or refuse HPV vaccination, there was not much I could say to change their minds” were example items. Before the analysis, a negatively worded item was reverse coded so that all responses went in the same direction, and the scores of the item responses were recoded using a range of 0 to 100 with 0=strongly disagree, 25=disagree, 50=neutral, 75=agree, and 100=strongly agree. Higher scores indicated that providers had greater expectations that their parental discussions would lead to vaccination. As with the self-efficacy scale, these items were developed using cognitive interviews, but the authors did not report on the psychometric properties [20], and we chose this measure because there were no other validated measures for outcome expectations. In our sample, the outcome expectations scale demonstrated acceptable internal consistency with Cronbach alpha=.65.

#### Confidence in Human Papillomavirus Vaccine Safety

Confidence in the HPV vaccine safety was measured at the provider level using a single ordinal item created by the research team asking: “Last year **,** how confident were you personally in the safety of the HPV vaccine for preventing cancer?” Response options were on a 5-point Likert scale ranging from very low to very high, and, before the analysis, the item responses were recoded using a range of 0 to 100 (0=very low, 25=somewhat low, 50=neutral or not sure, 75=high, and 100=very high). Higher scores indicated greater levels of confidence in the HPV vaccine safety. For a multivariate analysis, the variable was dichotomized to compare very high with other categories.

#### Confidence of Human Papillomavirus Vaccine Effectiveness

This was measured at the provider level with a single ordinal item created by the research team that asked: “Last year **,** how confident were you personally in the effectiveness of HPV vaccine for preventing cancer?” Responses on a 5-point Likert scale ranged from very low to very high, and the scores were recoded using a range of 0 to 100 with 0=very low, 25=somewhat low, 50=neutral or not sure, 75=high, and 100=very high, as with the other scales. Higher scores mean greater levels of confidence in HPV vaccine effectiveness. This variable was dichotomized to compare very high with other categories for multivariate analysis.

#### Size of Clinic

This is a continuous variable at the clinic level, which represents the total number of providers, as reported by each clinic. Providers included physicians, NPs, and PAs in each clinic.

#### Location of Clinic

In total, 2 categorical variables at the clinic level were categorized based on the address of each clinic and 2 different US Census Bureau designations that reflect degree of urbanization. A metropolitan statistical area (MSA) is a geographic area that is associated with a least one urbanized area that has a population of at least 50,000 [24]. Non-MSAs are all areas outside of the designated MSAs. In addition, the US Census Bureau also defines 2 types of urban areas, which represent a densely settled group of Census tracts with a population meeting one of the following criteria: (1) 50,000 or more (urbanized area) or (2) at least 2500 and less than 50,000 (urbanized cluster) [24]. Each clinic was assigned values for the 2 separate variables based on the physical address as follows: (1) MSA versus non-MSA and (2) town/rural area (urbanized cluster) versus city (urbanized area).

### Outcome

#### Perceived Parental Hesitancy by Providers

This is the primary outcome variable for this study. For this variable, we calculated a sum score from 7-items representing possible parental concerns, for which the providers rated how much they thought each one was a barrier to immunizing their patients against HPV (eg, parental concerns about HPV vaccine safety and parental mistrust of vaccines in general). The responses for each item were on a 4-point Likert scale ranging from not a barrier at all to a major barrier. Before the analysis, the scores of the individual items were recoded by using a 0 to 100 range with 0=not a barrier at all, 33=a minor barrier, 67=somewhat of a barrier, and 100=a major barrier, for consistency with the other scales that were measured using 4-point Likert scales. Higher scores represented greater levels of perceived parental hesitancy. This measure was adopted from Farias et al (2017) [19]. The authors did not report on the process used to develop the items or the psychometric properties of the scale. We selected this measure as we could not locate any other validated measures for perceived parental hesitancy for providers. In our sample, the provider-perceived parental hesitancy scale demonstrated good internal consistency with Cronbach alpha=.73.

#### Covariates

**Figure 1:**
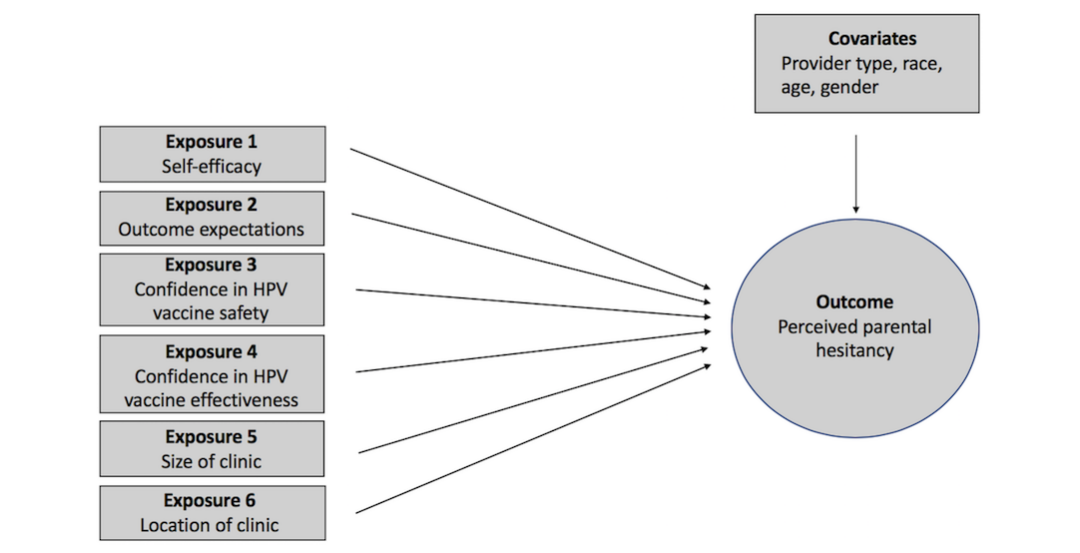
Depiction of variables. HPV: human papillomavirus.

Provider age was a continuous variable measured in years. Providers self-identified their race/ethnicity as White, Black, Hispanic, Asian, or other. To create a dichotomous variable for race for this analysis, Black, Hispanic, Asian, and other were combined in the category *nonwhite*, because of low number of participants in these categories. Provider gender was represented with the categories of male and female. Provider type included physician, NP, or PA. For this study, NPs and PAs were combined into 1 category as nonphysician providers because only 3 PAs were in this dataset. Years of provider experience was not included as a covariate because it was highly correlated with age (Pearson’s *r*=0.90, *P*<.001; results not shown). Figure 1 depicts the relationship between all of the variables.

### Statistical Analysis

#### Provider Characteristics by Provider Type

First, for descriptive purposes, provider-level demographic characteristics (age, race, and gender) were summarized for the overall sample and compared by provider type (ie, physicians and NPs/PAs) using Wilcoxon rank-sum test for continuous variables and Fisher’s exact test for categorical/binary variables. Continuous variables were summarized with median and quartiles, and categorical variables were summarized with frequencies and proportions.

#### Reasons for Perceived Parental Human Papillomavirus Vaccine Hesitancy by Provider Type

Second, for descriptive purposes, each of the individual items representing provider-perceived barriers of parental HPV vaccine hesitancy was summarized using frequencies and proportions, and also compared by provider type using Wilcoxon rank-sum test.

#### Perceived Human Papillomavirus Vaccine Hesitancy Scale and Exposure Variables by Provider Type

Next, the outcome variable (ie, perceived HPV vaccine hesitancy scale) and the exposure variables were summarized for the total sample with median and quartiles for continuous variables, and with frequencies and proportions for categorical variables. The outcome and exposures were compared by provider type, using Wilcoxon rank-sum test and Fisher’s exact test.

#### Linear Regression Analysis for Perceived Parental Human Papillomavirus Vaccine Hesitancy by Provider-Level Factors and Clinic-Level Factors

The objective of the primary analysis was to identify provider- and clinic-level factors associated with parental HPV vaccine hesitancy as perceived by providers. To estimate the associations between the outcome and the provider-level exposure variables of interest (ie, self-efficacy, outcome expectation, confidence in HPV vaccine efficacy, and confidence in HPV vaccine safety), a multilevel linear regression model was fit, taking one exposure variable at a time. We used linear mixed-effects models to account for clustering at the clinic level (ie, correlations between observations from the same clinic) through a random effect. Due to small numbers of responses in some of the categories, confidence in efficacy and confidence-in-safety variables were dichotomized into v*ery high* and o*ther*. The model also included age, race, gender, and type (physician NP/PA) of the providers. To investigate the clinic-level characteristics and their association to the primary endpoint, a similar multilevel model was fit with additional clinic-level variables: size and location. Clinic size was represented by the total number of providers and location was categorized into urban/rural. The provider-level variables (ie, age, race, gender, and type) were also included in this model. A significance level of alpha=.05 was selected. R version 3.5 by the R Foundation was used for all statistical analyses [25].

## Results

### Provider Characteristics by Provider Type

The survey was sent to all 187 providers located within the 23 clinics. Of these, 137 completed the survey, and all were used in this analysis except for 1 participant that did not finish the survey and had incomplete data. Table 1 provides demographic characteristics of the population. The median age of the 98 physicians was 47.0 (Quartiles: 42.0-52.0) and 33.0 (Quartiles: 28.2-39.8) for the 38 NPs/PAs. Among physicians, the majority were white (85%) and female (61%). Among NPs and PAs, almost all were white (97%) and female (95%). Overall, there were significant differences by provider type for age (*P*<.001) and gender (*P*<.001; Table 1). As it relates to a clinic location, there were 18 clinics in urban areas and 5 clinics in rural areas. For clinic size, the overall median was 5 providers (Quartiles: 4.0-6.5; Results not shown).

**Table 1:** Provider characteristics at baseline by provider type.

Variable		All providers (N=136)	Physicians (n=98)	NPs^a^/PAs^b^ (n=38)	*P* value
Age (LQ^c^-UQ^d^), years		45.0 (35.0-51.0)	47.0 (42.0-52.0)	33.0 (28.2-39.8)	<.001
**Race, n (%)**					**.07**
	White	119 (88)	83 (85)	36 (97)	—^e^
	Nonwhite	16 (12)	15 (15)	1 (3)	—
**Gender, n (%)**					**<.001**
	Male	40 (29)	38 (39)	2 (5)	—
	Female	96 (71)	60 (61)	36 (95)	—

^a^NPs: nurse practitioners.

^b^PAs: physician assistants.

^c^LQ: lower quartile.

^d^UQ: upper quartile.

^e^Not applicable.

### Reasons for Perceived Parental Human Papillomavirus Vaccine Hesitancy by Provider Type

The individual items representing reasons for parental HPV vaccine hesitancy as perceived by provider type are described in Multimedia Appendix 1. Among all providers, the majority reported the following as perceived barriers (ie, combination of somewhat of a barrier and a major barrier categories): parental concerns of HPV vaccine safety (88%), parental belief in child is too young for HPV vaccine (78%), parental belief that child is not at risk of HPV infection through sexual contact (70%), and parental mistrust in vaccines in general (59%). Yet, the least commonly reported barriers (ie, combination of somewhat or major barrier categories) were parental concern of getting too many shots during a visit (ie, HPV, Tdap, and Meningococcal; 40%), parental concerns about HPV vaccine efficacy (30%), and parental concerns about out-of-pocket costs (13%). Similar results were found among physicians versus NPs/PAs, except for significant differences in perception toward being too young to get the vaccine (81% vs 73%; *P*=.04) and parental concern regarding their child getting too many shots during a visit (44% vs 9%; *P*=.003), with physicians being more likely to perceive these as somewhat or a major barrier than NP/PA.

### Perceived Human Papillomavirus Vaccine Hesitancy and Exposure Variables by Provider Type

Table 2 summarizes the outcome variable (perceived HPV vaccine hesitancy) and the exposure variables for total sample and by provider type. For perceived barriers to HPV vaccination (outcome variable), the median score for all providers was 52.0, with physicians having greater perceived barriers than NPs/PAs (*P*=.009). The exposures did not differ by provider type. For all providers, the median score was 75.0 for *self-efficacy* and 62.0 for *outcome expectations*. Among physicians, the majority had high to very high confidence in the effectiveness of the HPV vaccine (95%) and safety of the HPV vaccine (97%). Similarly, NPs/PAs had high to very high confidence in the effectiveness of the HPV vaccine (92%) and safety of the HPV vaccine (89%).

**Table 2:** Outcome and exposure variables by pediatric provider type.

Variable		All providers (N=136)	Physicians (N=98)	NPs^a^/PAs^b^ (N=38)	*P* value
Outcome: perceived parental hesitancy (LQ^c^-UQ^d^)		52.0 (43.0-62.0)	57.0 (43.0-67.0)	48.0 (38.0-57.0)	.009
**Exposures, (LQ-UQ)**					
	Self-efficacy (Q)	75.0 (62.0-88.0)	75.0 (62.0-88.0)	71.0 (60.0-88.0)	.43
	Outcome expectations (Q)	62.0 (50.0-69.0)	62.0 (50.0-69.0)	56.0 (50.0-69.0)	.89
**Confidence: HPV^e^ vaccine effectiveness, n (%)**					**.52**
	Very/somewhat low	1 (1)	0 (0)	1 (3)	—^f^
	Neutral or not sure	7 (5)	5 (5)	2 (6)	—
	High	41 (31)	30 (31)	11 (31)	—
	Very high	85 (63)	63 (64)	22 (61)	—
**Confidence: HPV vaccine safety, n (%)**					**.23**
	Very/somewhat low	1 (1)	0 (0)	1 (3)	—
	Neutral or not sure	6 (5)	3 (3)	3 (8)	—
	High	46 (35)	34 (35)	12 (33)	—
	Very high	80 (60)	60 (62)	20 (56)	—

^a^NPs: nurse practitioners.

^b^PAs: physician assistants.

^c^LQ: lower quartile.

^d^UQ: upper quartile.

^e^HPV: human papillomavirus.

^f^Not applicable.

### Linear Regression Analysis for Perceived Parental Human Papillomavirus Vaccine Hesitancy by Provider-Level Factors and Clinic-Level Factors

Table 3 reports the associations between the provider’s perceived parental HPV vaccine hesitancy score and provider’s *self-efficacy*, *outcome expectations*, *confidence in vaccine effectiveness*, and *confidence in vaccine safety* based on the mixed-effects models, adjusting for age, race, gender, and provider type. A 10-point increase in *self-efficacy* was associated with a 2.9-point (95% CI 1.2-4.7) decrease in perceived parental HPV vaccine hesitancy when adjusting for covariates (*P*=.001). Similarly, a 10-point increase in *outcome expectations* was associated with a 3.7-point (95% CI 1.8-5.8) decrease in perceived parental HPV vaccine hesitancy (*P*<.001). For *confidence in HPV vaccine safety*, participants who had *very high* confidence on average scored 4.8 points lower on the perceived parental HPV vaccine hesitancy scale than the participants who had lower levels of confidence (*P*=.009). No significant association was found between perceived parental HPV vaccine hesitancy and *confidence in HPV vaccine effectiveness*. In all models, age, race, gender, and type of provider were not found to be significantly associated with the outcome, except the confidence-in-safety model, where female providers had a higher perceived parental HPV vaccine hesitancy by 6.9 points (95% CI 0.6-13.0; *P*=.03; results not shown).

Table 4 reports the associations of *clinic size* and *location* (town/rural vs city and non-MSA vs MSA) with perceived parental HPV vaccine hesitancy score. Including clinic-level variables in the model revealed no significant association between the clinic-level characteristics and perceived parental HPV vaccine hesitancy. Although adjusting for provider characteristics (age, gender, race, and type) and clustering by clinic, neither clinic size (*P*=.92) nor town/rural (*P*=.87) or non-MSA (*P*=.56) was found to be significantly associated with the outcome.

**Table 3:** Association between perceived parental human papillomavirus vaccine hesitancy score and provider level exposures using mixed-effect model (the mixed-effects models included one study exposure at a time and adjusted for age, race, gender, and provider type).

Provider-level factors	Estimate	Standard error	*t* value	*P* value
Self-efficacy	–0.29	0.08	–3.43	.001
Outcome expectations	–0.37	0.09	–4.05	<.001
Confidence in vaccine effectiveness: very high versus other (ref)	–5.20	2.88	–1.80	.07
Confidence in vaccine safety: very high versus other (ref)	–4.8	2.77	–1.72	.009

**Table 4:** Association between perceived parental vaccine hesitancy score and clinic-level exposures (the model includes one study exposure and adjusts for age, race, gender, and type of the providers).

Clinic-level factors		Estimate	Standard error	*t* value	*P* value
Clinic size		0.11	1.12	0.10	.92
**Clinic location**					
	Town/rural versus city (ref)	1.06	6.46	0.17	.87
	Non-MSA^a^ versus MSA (ref)	–4.53	7.68	–0.59	.56

^a^MSA: metropolitan statistical area.

## Discussion

### Principal Findings

We characterized perceived reasons for parental HPV vaccine hesitancy as perceived by pediatric providers. Not surprisingly, we found that the majority of providers perceived HPV vaccine safety, mistrust in vaccines, low perceived risk for HPV via sexual contact, and child’s young age as major parental barriers to HPV vaccination. A handful of previous studies have sought to characterize perceived barriers to HPV vaccine hesitancy according to providers [19,20]. McRee et al (2014) found that child not being sexually active, perception of child not being susceptible to an HPV-related disease, discomfort with sex talks with their child, and concerns about vaccines in general as the most common perceived reasons of parental HPV vaccine hesitancy among providers in Minnesota in 2013 [20]. Although providers in our sample also reported some of these reasons for parental hesitancy, safety concern about the HPV vaccine was the top reason in our sample. This could represent a difference across states or a shift in hesitancy reasons over the 5 years since that survey was conducted. Provider concern about vaccine safety was associated with lower patient vaccination uptake according to Farias et al (2017) [19]. As provider-perceived barriers have been found to contribute to lower HPV vaccine uptake [19,21], further research is needed on a larger scale, statewide and nationally, to monitor perceived parental barriers according to providers and by type. This will continue to inform intervention targets and establish generalizability of our findings. In addition, future studies should identify if providers are overestimating and misinterpreting reasons for parental hesitancy compared with actual parent-reported sources of parental hesitancy to be used as intervention targets.

This study was the first to identify if perceived reasons for parental HPV vaccine hesitancy varied by the type of pediatric provider. We observed significant differences in perceived barriers (ie, concern of their child getting too many shots during a visit and being too young to get the vaccine) by provider type. These findings suggest parental perceived barriers can vary across providers, with physicians more likely to perceive these as a major barrier. A possible explanation for this variation could be if the type of educational training related to vaccine hesitancy differs between physicians and other providers. In addition, physicians may have experienced different interactions with parents compared with other providers, which may lead to differences in perceived parental barriers. Hence, tailored strategies or messages by provider type could be used in interventions to assist them in addressing HPV vaccine parents.

In this sample, the majority of the providers had high to very high levels of confidence in HPV vaccine safety and effectiveness, as well as high levels of outcome expectations and self-efficacy. The level of outcome expectations and self-efficacy was similar to another study [20], which shows that providers still have room to improve on these factors. A previous study found that providers with higher levels of self-efficacy and outcome expectations had more routine recommendations of the HPV vaccine and increased ability to address hesitant parents [20]. Due to the role these factors play in providers perceiving lower parental hesitancy, interventions aimed at training providers on how to address HPV vaccine hesitancy may benefit from targeting provider confidence in safety, self-efficacy, and outcome expectations.

To our knowledge, this is the first study to also identify provider and clinic-level factors associated with perceived parental barriers to HPV vaccine hesitancy according to providers. One explanation for the positive association between perceived parental barriers and self-efficacy, outcome expectations, and confidence in vaccine safety could be that providers who engage in unsuccessful encounters with HPV vaccine hesitant parents increase their perceived parental barriers to HPV vaccination while lowering their self-efficacy and outcome expectations. Furthermore, providers with low confidence in HPV vaccine safety may unintentionally transfer their own uncertainty to the parents of their patients and perceive that parents are more hesitant than they actually are [7].

Surprisingly, in viewing clinic-level factors associated with perceived provider barriers, clinic location was not found statistically significant. In 2017, rural areas had lower coverage rates for receipt of the first dose of the HPV vaccine compared with urban areas, with a difference of 11 percentage points [26]. This raises the question of whether HPV vaccination is lower in rural areas because parental hesitancy is more prevalent, or because providers are less likely to recommend the vaccine or recommend it effectively in rural areas due to perceived parental barriers to HPV vaccination. As physicians play a key role in parental acceptance, the lack of association here warrants more research to gain a better understanding of the factors influencing physician recommendations in rural areas.

### Limitations

This study was not without limitations. First, this was a cross-sectional study with a “snapshot” captured of factors influencing perceived parental HPV vaccine hesitancy according to pediatric providers. Therefore, we could only examine associations and not causality. Perceived provider hesitancy could also influence the independent variables, and given the cross-sectional and correlational design, we could not test direction of causality. Nevertheless, this study contributes valuable information to the literature as the first to examine this question, and future research is needed to explore potential bidirectional relationships with the outcome variable using a longitudinal design or more complex relationships using a qualitative research. Second, we had a convenience sample of clinics and providers in Tennessee who participated in the larger study in 2018. Thus, the sample was limited to a subset of the pediatric provider population (ie, primarily white and female). The specific context of time and place could limit the generalizability of results to other regions of the country, given that Tennessee has relatively low levels of HPV vaccination; furthermore, perceived parental vaccine hesitancy could change over time as vaccination coverage increases. In addition, we were unable to test for differences in perceived barriers to HPV vaccination between NPs and PAs because of the small sample size. However, this was the first study to examine this question and will inform future studies in larger samples that can test this comparison. The measures (ie, self-efficacy, outcome expectations, and perceived parental hesitancy) adopted from previous studies were unvalidated at the time of study; however, we found them to have acceptable reliability. We included all of the demographic variables from the survey as covariates except for years of experience of the providers because of its strong correlation with age; we were unable to account for other potential unmeasured confounders. Given that the data were self-reported, there was potential for social desirability and recall bias. Finally, because this study only surveyed providers and not parents, we could not assess how well the perceived reasons of the providers aligned with actual reasons for hesitancy of the parents. This study points to the need for future research to do so.

### Conclusions

Provider perceptions of parental barriers to HPV vaccine hesitancy are an important factor contributing to HPV vaccine uptake among parents [19,21]. Their perceptions of parent barriers may influence their likelihood to recommend the vaccine and how they communicate the recommendation [19], which is a major issue as their recommendation is the strongest predictor of HPV vaccine uptake [27,28] and parents prefer a strong provider recommendation [13,14]. Strategies are needed to effectively reduce provider-perceived barriers to parental HPV vaccine hesitancy and to assist providers in addressing these barriers in patient-provider communication. Our results suggest intervention targets to improve provider perceptions of parental barriers by addressing specific factors that may influence their perceptions. Particularly, intervention developers should consider addressing providers’ self-efficacy, perceived outcome expectations, and confidence in HPV vaccine safety. Ultimately, addressing these provider-level factors may improve recommendation practices and communication strategies among providers for addressing hesitancy, to increase HPV vaccination rates among children of HPV vaccine hesitant parents.

## Appendix

Multimedia Appendix 1 Reasons for parental human papillomavirus vaccine (HPV) hesitancy as perceived by pediatric provider type.

## Abbreviations

CPF: Cumberland Pediatric Foundation
HPV: human papillomavirus
MSA: metropolitan statistical area
NP: nurse practitioner
PA: physician assistant
