# Supplementary material for: Providers’ Perceptions of Parental Human Papillomavirus Vaccine Hesitancy: Cross-Sectional Study
Source: JMIR Cancer. 2019 Jul 2;5(2):e13832. doi: 10.2196/13832 (PMC6632100; doi:10.2196/13832)
Supplement: Multimedia Appendix 1 [file cancer_v5i2e13832_app1.pdf]

Multimedia Appendix. Reasons for parental human papilloma virus (HPV) vaccine hesitancy as perceived by pediatric provider type.

Variable		All Providers (N=136)	Physicians (N=98)	NPs/PAs[a] (N=38)	*P* value
**Parental concerns: HPV vaccine safety, n (%)**					
	Not a barrier at all	3 (2)	3 (3)	0 (0)	.53
	A minor barrier	13 (10)	8 (8)	5 (14)	—[b]
	Somewhat of a barrier	57 (43)	40 (41)	17 (47)	—
	A major barrier	60 (45)	46 (47)	14 (39)	—
**Parental belief: child is too young for HPV vaccine, n (%)**					
	Not a barrier at all	8 (6)	5 (5)	3 (8)	.04
	A minor barrier	21 (16)	14 (15)	7 (19)	—
	Somewhat of a barrier	57 (43)	37 (39)	20 (56)	—
	A major barrier	46 (35)	40 (42)	6 (17)	—
**Parental belief: child is not at risk for HPV infection through sexual contact, n (%)**					
	Not a barrier at all	10 (8)	6 (6)	4 (11)	.20
	A minor barrier	30 (23)	19 (20)	11 (31)	—
	Somewhat of a barrier	55 (42)	40 (42)	15 (42)	—
	A major barrier	37 (28)	31 (32)	6 (17)	—
**Parental mistrust: vaccines in general, n (%)**					
	Not a barrier at all	5 (4)	4 (4)	1 (3)	.99
	A minor barrier	49 (37)	36 (37)	13 (36)	—

	Somewhat of a barrier	59 (44)	42 (43)	17 (47)	—
	A major barrier	20 (15)	15 (15)	5 (14)	—
**Parental concern: getting HPV, Tdap, and Meningococcal is too many shots for the same visit, n (%)**					
	Not a barrier at all	22 (17)	9 (9)	13 (36)	.03
	A minor barrier	58 (44)	44 (45)	14 (39)	—
	Somewhat of a barrier	40 (30)	32 (33)	8 (22)	—
	A major barrier	13 (10)	12 (12)	1 (3)	—
**Parental concerns: HPV vaccine efficacy, n (%)**					
	Not a barrier at all	41 (31)	31 (32)	10 (28)	.79
	A minor barrier	52 (39)	35 (36)	17 (47)	—
	Somewhat of a barrier	30 (23)	23 (24)	7 (19)	—
	A major barrier	9 (7)	7 (7)	2 (6)	—
**Parental concerns: out-of-pocket costs for HPV vaccine, n (%)**					
	Not a barrier at all	64 (49)	41 (43)	23 (64)	.14
	A minor barrier	51 (39)	42 (44)	9 (25)	—
	Somewhat of a barrier	14 (11)	10 (11)	4 (11)	—
	A major barrier	2 (2)	2 (2)	0 (0)	—

[a]NP/PA: nurse practitioners/physician assistant.

[b]Not applicable.
